# Single-cell Raman spectroscopy detects pediatric focal cortical dysplasia

**DOI:** 10.1117/1.BIOS.2.1.015002

**Published:** 2025-02-18

**Authors:** Trang Tran, Frederick Dallaire, Joshua Sonnen, Romain Cayrol, Frédéric Leblond, Roy W. R. Dudley

**Affiliations:** a Polytechnique Montréal, Department of Engineering Physics, Montréal, Quebec, Canada; b Centre de Recherche du Centre Hospitalier Universitaire de l’Université de Montréal (CRCHUM), Imaging and Engineering Axis, Montréal, Quebec, Canada; c McGill University Health Center (MUHC), Department of Pathology, Montréal, Quebec, Canada; d Centre Hospitalier Universitaire de l’Université de Montréal (CHUM), Department of Pathology, Montréal, Quebec, Canada; e McGill University Health Center (MUHC), Department of Neurosurgery, Montréal, Quebec, Canada

**Keywords:** epilepsy, focal cortical dysplasia, tissue optics, microscopy, Raman spectroscopy, machine learning

## Abstract

**Significance:**

Focal cortical dysplasia (FCD) type II is the leading cause of drug-resistant focal epilepsy in children. While surgical resection offers the only definitive cure, its success is hindered by the challenge of precisely identifying the lesion and its boundaries. Despite advancements in neuroimaging, FCD type II often remains elusive, complicating surgical planning and outcome optimization. Enhanced detection methods are crucial to improving the precision of resection and, ultimately, achieving seizure freedom in affected patients.

**Aim:**

Advanced techniques for detecting FCD type II margins during surgery are critically needed to enhance postoperative outcomes. Spontaneous Raman spectroscopy is a label-free optical method that allows the characterization of the tissue’s biochemical composition. The goal of this proof-of-concept study was to compare—in pediatric patients—the spectral signature of abnormal cells in FCD tissue with cells associated with the normal cortex.

**Approach:**

A Raman microspectroscopy imaging workflow was developed and applied to 70 surgical specimens from 30 focal epilepsy patients diagnosed with FCD type II. Raman spectra from individual cells were recorded from FCD type II specimens (dysmorphic neurons and balloon cells) and normal brains (neurons). Machine learning models (support vector machines) were trained, validated, and tested to distinguish FCD tissue from the normal brain as well as to distinguish between two disease subtypes, i.e., FCD types IIa and IIb.

**Results:**

A total of 1420 single-cell spectra were acquired and spectral differences determined between FCD type II and normal cortex, as well as between FCD type IIa and type IIb. Machine learning distinguished FCD type II from the normal cortex with 96% accuracy, 100% sensitivity, and 95% specificity. FCD types IIa and IIb specimens were distinguished with 92% accuracy, 100% sensitivity, and 86% specificity.

**Conclusions:**

The Raman spectroscopy signature of single cells associated with FCD tissue was established. This provides credence to the hypothesis that Raman spectroscopy as a technique—if implemented using a fiber optics system—has the potential for safely optimizing the extent of FCD type II resection in pediatric focal epilepsy surgery. In addition, this technique provides insights into multiple biochemical alterations within dysplastic tissues, which may contribute to the underlying mechanisms of epileptogenesis.

Statement of Discovery This work utilizes Raman microspectroscopy to detect pediatric focal cortical dysplasia. The technique offers insights into the biochemical alterations within dysplastic tissues, shedding light on the mechanisms underlying epileptogenesis, toward enhanced surgical precision in pediatric epilepsy cases.

## 1 Introduction

More than 50 million people worldwide suffer from epilepsy with approximately half being children.^1^ Over one-third of this population does not respond to medications, leaving surgical resection the only option for seizure control. Seizure onset is focal for 60% of all drug-refractory cases, and surgery can be curative when the epileptogenic zone (EZ) is removed completely.^2^^,^^3^ The most common cause of focal epilepsy in children is focal cortical dysplasia (FCD), with FCD type II being the most prevalent.^2^ FCD is classified into different neuropathological subtypes based on the degree of cytoarchitectural disruption visible in histology. In type I, the cortical layers are disorganized (dyslamination) without abnormal or dysmorphic cells.^4^ Type II is characterized either by large dysmorphic neurons without balloon cells (type IIa) or with balloon cells (type IIb). Type III exhibits cortical lamination abnormalities akin to type I, yet they are associated with other pathologies: hippocampal sclerosis (type IIIa), tumors (type IIIb), vascular malformations (type IIIc), and acquired lesions including strokes or previous trauma (type IIId).^5^

An FCD lesion is challenging to resect completely because it is often difficult to fully map its extent with confidence, even with the most advanced neuroimaging and electrophysiology methods.^6^ This is because the borders of the lesions are usually ill-defined, undetected on routine but only found in histopathology.^7^ As much as 40% to 60% of patients continue to have seizures after epilepsy surgery because of residual pathological tissue associated with abnormal epileptogenic cells, including dysmorphic neurons.^3^ Surgical adjuncts, such as intraoperative magnetic resonance imaging (iMRI) and electrocorticography (Ecog), have failed to improve postsurgical seizure outcomes because the FCD lesion borders are not visible on MRI and/or do not always produce interictal electrical discharges during intraoperative Ecog recordings.^8^ There is currently no intraoperative technique that can detect FCD or its borders with sufficiently high sensitivity and specificity to improve postsurgical seizure outcomes.

Raman spectroscopy has emerged as a promising technique to quantify the biomolecular composition of tissue *in vivo*^9^ and individual cells *ex vivo*^10^ The technique relies on the detection of inelastically scattered laser light that interacts with the vibrational modes of molecular bonds associated with common biomolecules, including proteins, specific amino acids, lipids, and nucleic acids.^11^ The molecular signature of tissue or cells can be detected non-invasively without labeling (i.e., no contrast agent nor staining).

The result is a unique molecular fingerprint—a Raman spectrum—from which pathological molecular features can be extracted by comparison with control measurements from normal tissue or normal cells. This comparison is usually achieved using machine learning techniques leading to the production of predictive mathematical models that can subsequently be used for automated pathology detection.^9^

Clinical applications of Raman spectroscopy have gained traction as a method to detect cancer cells in a wide range of human pathologies.^10^ Most recently, Raman spectroscopy has shown value as an intraoperative method to detect residual cancer at surgical margins. For example, an intraoperative single-point Raman spectroscopy probe was developed by our group for glioblastoma surgery guidance with the objective to improve tumor resection safety and maximize the volume of resected cancer.^11^^,^^12^ This is important because glioblastoma are invasive tumors that are infiltrating the normal brain. No intraoperative techniques existed that allowed to detect cancer associated with low densities of cancer cells at a level consistent with infiltrations extending beyond the MRI-enhancing ring of a glioblastoma.^13^ There is a similar need for pediatric FCD type II lesions in epilepsy surgery. Currently, little has been studied in epilepsy surgery using Raman spectroscopy, particularly in human tissues. In only a single small series of 10 biopsy specimens from pediatric patients undergoing focal epilepsy surgery, Anand et al.^14^ found that the technique could distinguish seven heterogeneous FCD specimens from three normal specimens with 100% sensitivity and 90% specificity.

This work presents the first building blocks toward a comprehensive understanding of FCD types IIa and IIb. Raman microspectroscopy was used to interrogate single cells from FCD or normal tissue specimens, either dysmorphic neurons, balloon cells, or normal neurons. Machine learning models were trained, validated, and tested that could discriminate normal from dysplastic tissue. This work sets up the stage for the future development of intraoperative techniques, using a hand-held Raman spectroscopy probe, to be used toward detecting the whole extent of FCD lesions live during surgery. This will be of paramount importance to safely optimize the removal of epileptogenic tissue and give the best chance of postoperative seizure freedom.

## 2 Materials and Methods

### 2.1 Tissue Specimens and Pre-Imaging Preparation

**Fig. 1:**
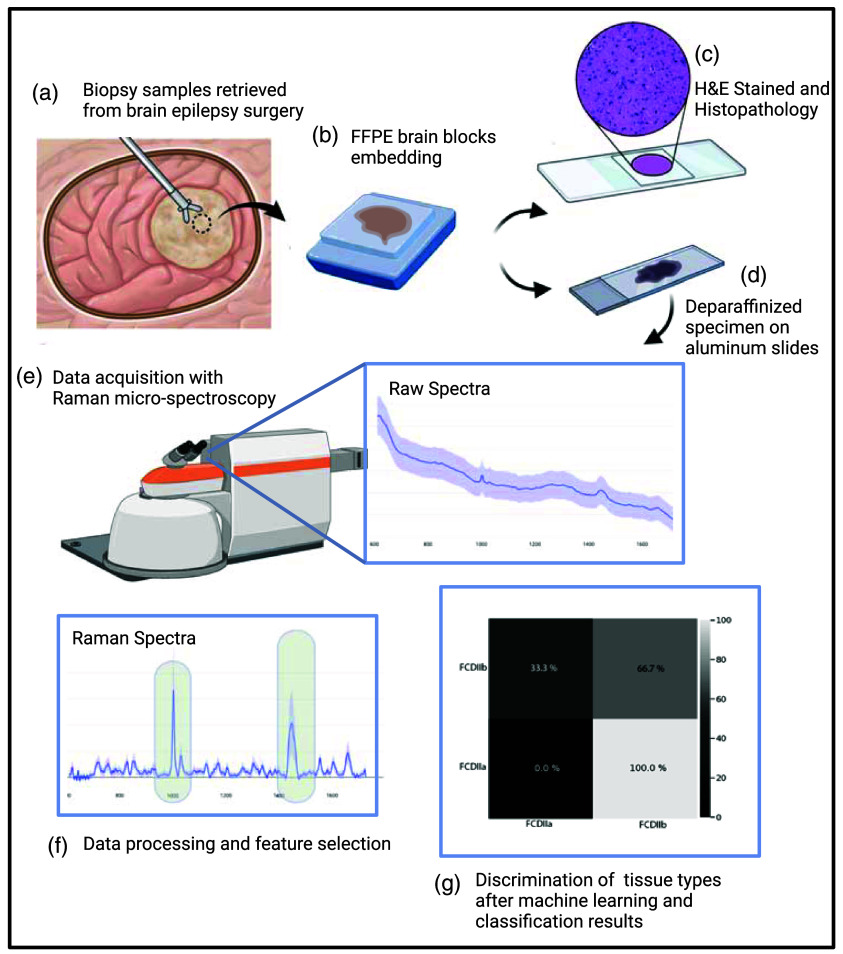
Schematic representation of the Raman microscopy acquisitions on biopsy samples to the statistical analysis and classification model.

Seventy formalin-fixed paraffin-embedded (FFPE) biopsy specimens were obtained from 30 patients at the Montreal Children’s Hospital. The study was approved by the McGill University Health Center (MUHC) institutional research ethics board. Patients/parents signed an informed consent allowing the investigative use of the specimens. The median age of the patients was 8±3.30 years. Twelve patients were males, and 18 patients were females: 16 had left frontal lobe epilepsy, 11 had right frontal lobe epilepsy, 2 had left temporal lobe epilepsy, and 1 had right temporal epilepsy. All patients were included in at least two antiseizure medication trials, and their symptoms could not be controlled with drugs. Forty specimens were associated with FCD type II of which 21 were type a and 19 were type b. The remaining 30 biopsy specimens were from the normal cortex resected as part of the regular surgical approach to the FCD lesion. Two adjacent sections were cut from the FFPE blocks. One section with a width of 4  μm was used for Raman microspectroscopy imaging, and the other was stained with hematoxylin and eosin (H&E) for histopathological analyses (Fig. 1). The H&E sections were analyzed by board-certified neuropathologists involved in the study (J. S. and R. C.).

A deparaffinization protocol was applied to the sections dedicated to spectroscopy analyses. This consisted of (1) washing in xylene (three washes of 1 min each), (2) washing in 100% ethanol (two washes of 1 min each), (3) washing in 95% ethanol (two washes of 1 min each), (4) washing in 70% ethanol (two washes of 1 min each), and (5) washing in 50% ethanol (two washes of 1 min each). The slices were then placed on an aluminum slide and left to dry overnight. Aluminum was selected because of its low Raman activity, to minimize signal contamination.^15^

### 2.2 Raman Microspectroscopy Cellular Imaging Protocol

**Fig. 2:**
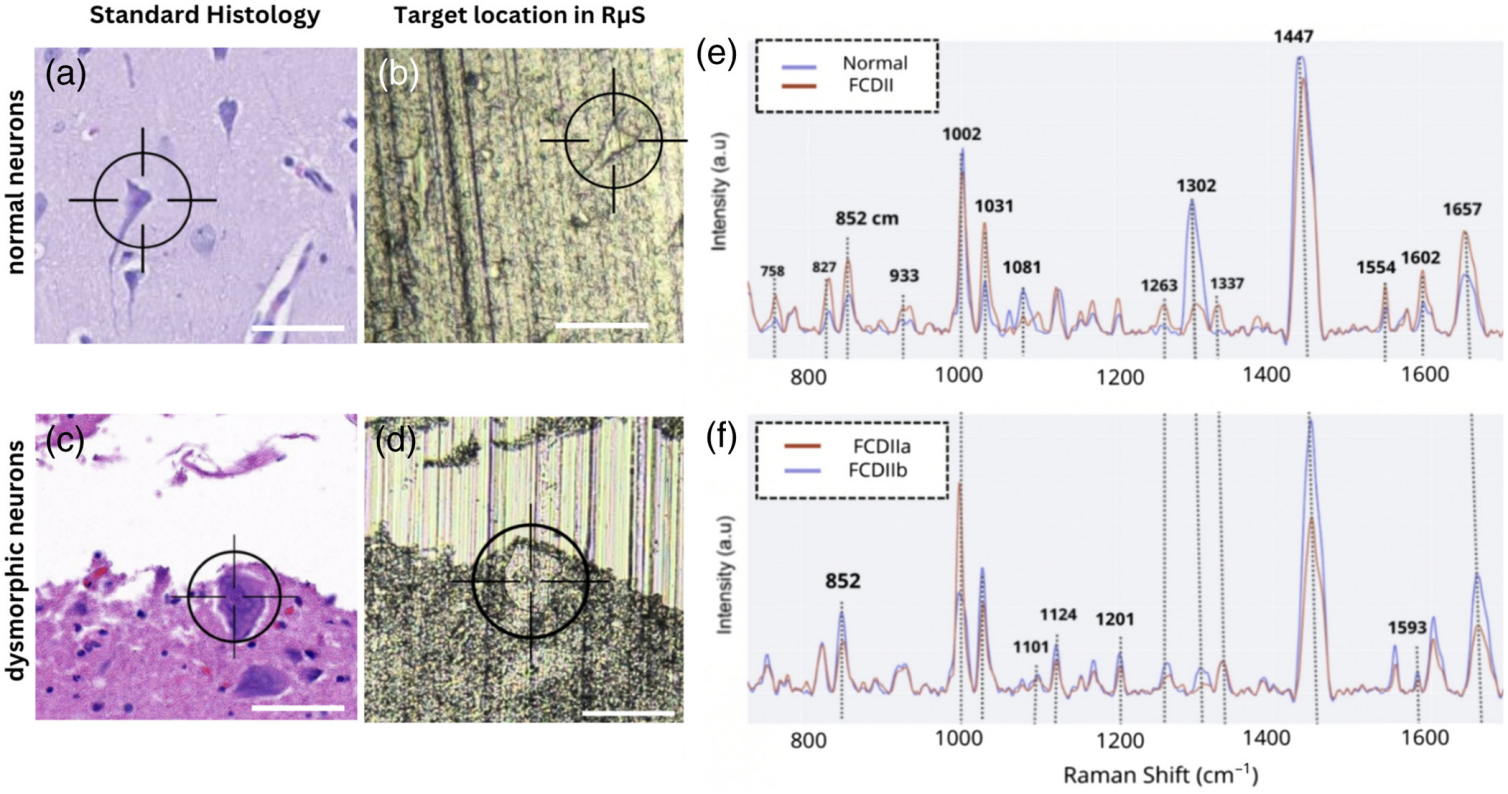
Raman spectroscopy of normal brain compared with dysplastic tissue in a single-cell analysis. H&E-stained sections of (a) normal neurons, (c) dysmorphic neurons, (b) target location of normal neurons under the Raman microscope, and (d) dysmorphic neurons. (e) Average spectra from FCD type II (in red) and normal brain (in blue). (f) Average spectra from FCD type IIa in red and FCD type IIb in blue. Scale bar—20  μm.

Raman microspectroscopy measurements were carried out using the inVia confocal Raman microscope (Renishaw, Gloucestershire, United Kingdom). The excitation wavelength was 785 nm with 40-mW laser power using the in-line focus mode, which corresponds specifically to the optical power measured at the sample plane (diffraction grating: 1200 lines per millimeter), and the spectral domain was 300 to $1800\;{\mathrm{cm}}^{-1}$ (1024 individual spectral bins). Each acquisition lasted 100 s (10 accumulations of 10 s) using a 50× short working distance objective that had a numerical aperture of 0.75. The 10 accumulations were averaged to maximize photonic count and limit stochastic noise. A feature of the microscope was that bright-field images were automatically collocated with the field-of-view accessible for Raman microspectroscopy measurements. This was used to locate cells and ensure spectroscopic measurements were made within cells and not in the extracellular matrix [Figs. 2(a)–2(d)]. Recognizable morphological structures, on the white-light image, were used to identify cells as either normal pyramidal neurons, balloon cells, or dysmorphic neurons. The cell type was further confirmed from the H&E slide collocated with the white-light image.

The final dataset had 700 normal neuron spectra from 35 normal cortex specimens and 770 spectra from 35 FCD type II specimens. In the latter category, 396 spectra were associated with FCD type IIa from 18 specimens, and 374 spectra were from 17 FCD type IIb specimens. FCD Type IIa samples were only associated with dysmorphic neurons, whereas FCD type IIa had both dysmorphic neurons and balloon cells (Table 1).

**Table 1:** Classification results associated with the two machine learning models. Model 1 was trained to distinguish. FCD type II from the normal brain while model 2 distinguished cells from FCD types IIa and IIb.

		No. of specimens (no. of spectra)	No. of spectra trained	No. of spectra tested	Sensitivity (%)	Specificity (%)	Accuracy (%)
Model 1	Normal	35 (700)	579	294	100	95	96
	FCDII	35 (770)	597				
Model 2	FCD type IIa	18 (396)	305	231	100	86	92
	FCD type IIb	17 (374)	308				

**Fig. 3:**
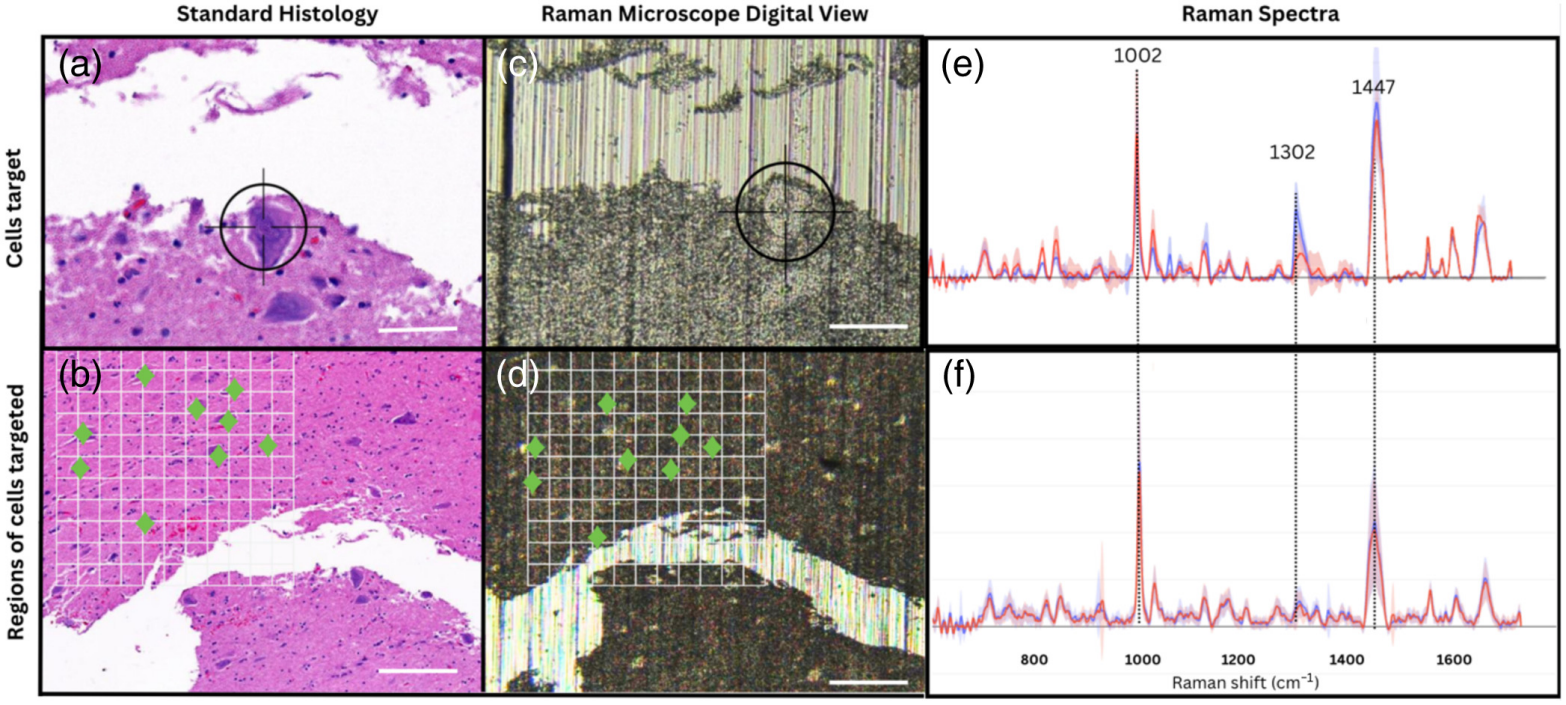
Target selection comprising dysmorphic neurons and the grid mapping scans represented by the white grid. (a) and (b) Mirror H&E digital file. Scale bar—20  μm. (c) and (d) Mapping neuronal composition with Raman microscopy via the digital viewer. Scale bar—80  μm. (e) Raman spectra average and the variance of FCD versus normal: the spectra in blue is the control tissue and the FCDII tissues in red. (f) Raman spectra average and the variance of FCDIIa versus FCDIIb: blue spectra are the FCDIIa, and red spectra are red the FCDIIb. The green icons represent the laser spot targeting in a specific region, aiming 7 to 10 cells per region.

To locate the specific cells (i.e., balloon cells, dysmorphic neurons, and normal neurons), bright-field montage images of the interrogated region were obtained with a 5×, 20×, and 50× lens (Fig. 3). To ensure the accuracy of a targeted neuron region on the microscope camera, the H&E digital file for the corresponding serial section annotated by the neuropathologist was used side by side to help locate it visually. Measures for proper quality control were taken when comparing Raman microscopy and the H&E digital file: wavelength (spectrometer) calibration, validating with a neuropathologist the accurate identification in the digital camera viewer of the Raman microscope, and the pathology department ensures a proper staining and image clarity in the H&E digital file for accurate cell characterization.

### 2.3 Spectral Data Pre-Processing and Dimensional Reduction

Standard data preprocessing steps were applied to the average spectroscopic measurement obtained for each cell:^16^ (1) subtraction of background signal associated with dark counts associated with a measurement performed with the laser turned off, (2) y-axis normalization to account for the instrument response using a NIST calibration standard, (3) x-axis (wavenumber axis) calibration using a measurement on an acetaminophen sample, (4) spectral smoothing using a Savitzky–Golay filter of order 3 with a window size of 11, (5) subtraction of the low-frequency background mostly attributable to intrinsic sample fluorescence using a custom adaptation of the rolling ball algorithm,^17^ (6) x-axis truncation restricted the spectral domain to the range 700 to $1700\;{\mathrm{cm}}^{-1}$ (900 spectral bins), and (7) standard normal variate (SNV) normalization and a spectral resolution of $1.1\;{\mathrm{cm}}^{-1}$.

### 2.4 Classification Models

A procedure was applied to each SNV-normalized Raman spectrum for dimensional reduction purposes.^18^ Specifically, a Gaussian fitting technique was applied to all peaks detected in each spectrum, outputting the peak position (in ${\mathrm{cm}}^{-1}$), its height, and its width (full-width-at-half-max). Overall, 24 peaks were consistently detected across the dataset, resulting in 72 spectral features. A feature selection procedure was applied to further reduce the number of features retained prior to training machine learning models.^18^ A linear support vector machine (SVM) with L1 regularization was used for that purpose, reducing the number of features to 13. Movasaghi et al.^19^ reported the most frequent Raman bands detected in biological tissue. Using this information, the resulting Raman features were identified, leading to a detailed biomolecular band assignment (Table 2).

**Table 2:** Comparison of principal Raman bands observed in normal/FCD and FCD type IIa/type IIb along with a tentative biomolecular assignment. Asterisks (*) are used to highlight peaks that are significantly different between different FCD and normal and FCD types IIa and IIb.

Raman peak (${\mathrm{cm}}^{-1}$)	FCD versus normal	FCDIIA versus FCDIIB	Main vibration mode	Molecule assignment
758	*		*ν*s (indole ring breathing)	Tryptophan
827	*		*ν*2 PO2—stretch	Nucleic acid
852	*	*	Tyrosine ring breathing and proline	Protein (collagen)
933	*		*ν*(C─C) skeletal of collagen backbone	Protein (collagen)
1002	*	*	*ν* ring breathing	Phenylalanine and protein
1031	*	*	δ(C─H) and bending mode	Phenylalanine and protein
1101		*	Amide III	Proteins
1124		*	*ν*(C─C) skeletal of acyl backbone in lipid (transconformation)	Lipid
1201		*	Amide III	Proteins
1263		*	Tring breathing modes of the DNA/RNA bases ═C─H bend (protein)	DNA/RNA
1302		*	δ(CH2) twisting, wagging, and phospholipid	Lipid and protein combination
1337		*	Ring breathing modes in the DNA bases	DNA
1447		*	CH2 deformation (protein vibration)	A marker for protein concentration
1554		*	*ν*(CN) and (NH) amide I	Protein
1593		*	C═C vibration	Retinoid and enzymes
1602			δ(C═C)	Phenylalanine and protein
1657		*	Amide I and fatty acids	Triglycerides

Another SVM was used to train a classification model from the reduced features using fivefold cross-validation. Two models were developed: FCD type II versus normal neurons (model 1) and FCD type IIa versus FCD type IIb (model 2). Model performance assessment was achieved using a receiver operating characteristic (ROC) analysis by comparing model predictions to their assigned pathology labels. A grid search procedure was applied for each model to determine optimal hyperparameters, i.e., C and the number of retained features. The optimal model was then tested on a testing set comprising 20% of the whole dataset randomly selected and set aside prior to training/validation. The ROC curve [along with the area under the curve (AUC)] was plotted for each model, and the confusion matrices were computed (Fig. 3). The sensitivity, specificity, and accuracy of detection of each model were reported corresponding to the parameter of the ROC curve associated with the point that was closest to the upper-left corner of the sensitivity versus (1−pecificity) graph (Table 1).

**Fig. 4:**
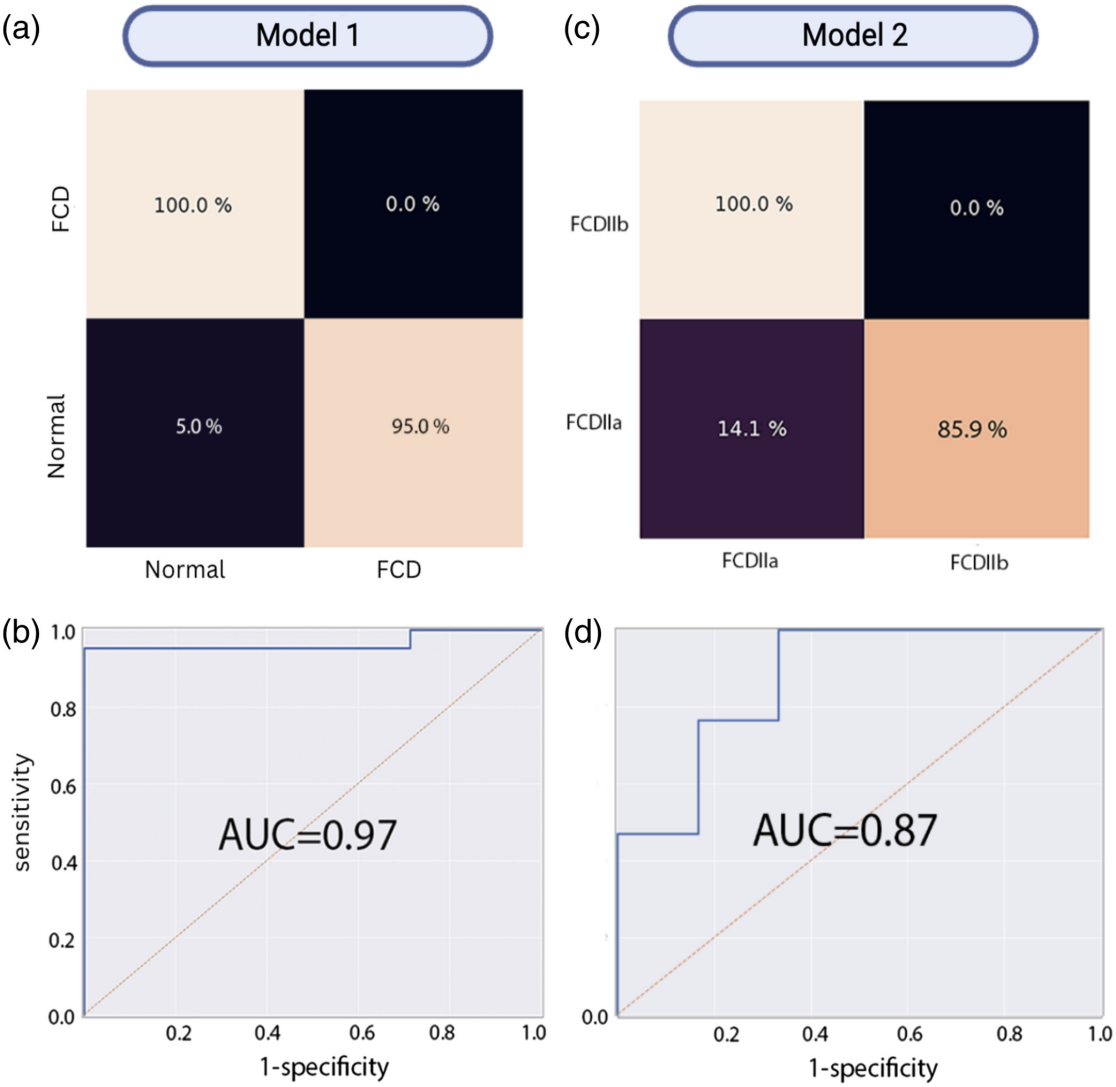
Confusion matrix heatmap and area under the ROC curve results of the two models. Model 1 was trained to distinguish FCD type II from the normal brain, whereas model 2 distinguished cells from FCD types IIa and IIb. Confusion matrix on a test out (a) normal versus FCD, (c) FCDIIa versus FCDIIb, and their respective ROC curve and AUC (b) and (d). The value in percentage represents the iterations of the test set. The higher the percentage, the lighter the color in the heatmap.

Using the features in Table 1, the machine learning model classified each of the testing tissues into normal or FCD. In Fig. 4, for each tissue type, the normal tissues were defined as control, and dysplastic tissues were defined as positive. The model was trained on 1200 spectra using a fivefold cross-validation with a mix of 4:1 ratio of FCD to control tissue samples; these results were externally validated on an unseen test set of 280 spectra with the same data distribution. The second model was trained on 640 spectra using a fivefold cross-validation with a mix of 4:1 ratio of FCD type IIa to FCD type IIb tissue samples, and these results were externally validated on an unseen test set of 160 spectra with the same data distribution.

## 3 Results

### 3.1 Raman Fingerprint of Dysplastic Tissue and Performance of the Machine Learning Models

The mean spectra of normal brain cells and those from FCD type II cells were compared [Fig. 2(e)]. FCD type II spectra had higher Raman peaks at 758, 827, 852, 933, 1263, 1337, 1554, 1602, and $1657\;{\mathrm{cm}}^{-1}$. On the other end, spectra associated with neurons from the normal cortex had higher peaks at 1002, 1031, 1302, and $1447\;{\mathrm{cm}}^{-1}$. A similar spectrum comparison was made between cells from FCD types IIa and IIb [Fig. 2(f)]. FCD type IIb had higher peaks at 852, 1031, 1101, 1124, 1201, 1263, 1302, 1447, 1554, and $1593\;{\mathrm{cm}}^{-1}$. FCD type IIa had a higher peak at $1002\;{\mathrm{cm}}^{-1}$.

Spectral features associated with the abovementioned Raman bands were used as the basis for training, validating, and testing the machine learning models. The predictive accuracy of the machine learning model trained to distinguish FCD type II cells from normal brain cells was 96%, with a sensitivity of 100% and a specificity of 95%. The ROC curve associated with that model had an AUC of 0.97. The predictive accuracy of the machine learning model trained to distinguish cells from FCD types IIa and IIa tissue was 92%, with a sensitivity of 100% and a specificity of 86%. The ROC curve associated with the models had an AUC of 0.87.

### 3.2 Biomolecular Interpretation of Pathology Changes

The Raman peaks that showed significant differences between FCD type II cells and normal brain cells were highlighted (Table 2, asterisks in the second column). The peaks that showed significant differences between FCD types IIa and IIb were also highlighted (Table 2, asterisks in the third column).

In the FCD type II specimens, a majority of the distinguishing peaks were linked to proteins, particularly phenylalanine and collagen. The presence and intensity of these peaks, such as the phenylalanine band at $1002\;{\mathrm{cm}}^{-1}$ and the protein band at $1447\;{\mathrm{cm}}^{-1}$, served as the standard markers for biological samples.^19^ This observation aligned with the findings by Anand et al.,^14^ who also noted similar protein peaks. Notably, in our study, peaks associated with amide III at 1101 and $1263\;{\mathrm{cm}}^{-1}$, as well as amide I at $1554\;{\mathrm{cm}}^{-1}$, were significantly higher in FCD type II specimens compared with normal brain tissue. Moreover, the discrepancy in intensity for these peaks was notably greater when comparing FCD types IIb to IIa. This heightened presence of amide groups was reminiscent of findings by Sacharz et al.,^20^ where increased amide content was reported in epileptic rat brain tissue analyzed using two-dimensional correlation Raman spectroscopy. This suggested a potential correlation between the intensity of amide peaks and the abundance of proteins in FCD type II specimens. Higher protein abundance typically corresponded to a greater number of peptide bonds and, consequently, a higher concentration of amide groups.^21^ This correlation was particularly evident in techniques such as Raman spectroscopy, where the intensity of amide bands served as an indicator of protein content in a sample.^22^ This association underscored the utility of amide bands in investigations aimed at elucidating abnormal protein aggregation-related pathologies or intricate biochemical processes.^23^

In addition to the protein-related insights provided by the analysis of amide bands, peaks corresponding to amino acids involved in DNA and RNA were also evident: 756, 827, 1263, and $1337\;{\mathrm{cm}}^{-1}$. These peaks provided valuable information about the genetic dysregulation underlying conditions such as FCD type II. Indeed, it has been shown that somatic mutations are causative in many cases of FCD type II.^24^ The most common of which appears to be a hyperactivation of the mTOR (mechanistic target of rapamycin) pathway, which is involved in neuronal migration and growth.^25^ Furthermore, Lee et al.^26^ and Baldassari et al. (2019) showed that the density of dysmorphic neurons and balloon cells in FCD type II brain specimens was positively correlated with the level of somatic mutation load. In Krochmalnek et al.,^27^ FCD specimens exhibiting clear histologic abnormalities demonstrated detectable pathogenic mutation variant loads, prompting inquiries into the existence of a tolerable variant threshold and the necessity for clean surgical margins, akin to tumor resections.

Two significant Raman bands were observed related to lipids: an increase at 1081 and $1302\;{\mathrm{cm}}^{-1}$ in normal tissues, and a higher intensity at $1124\;{\mathrm{cm}}^{-1}$ in FCD type IIb compared with FCD type IIa. Interestingly, our finding of an increase in the $1302\;{\mathrm{cm}}^{-1}$ band in normal tissue contradicts Anand et al.’s^14^ results, which showed elevated levels in dysplastic tissue from fresh specimens. However, our result aligned with Turker et al.’s^28^ hypothesis that lipid reduction during epileptic seizures is due to glutamate release activating phospholipases, which utilize membrane lipids as reservoirs and facilitate further glutamate release. It is important to note that in Raman spectra analysis, a single peak may not exclusively represent the lipid signal and could be attributed to a protein–lipid interaction. Therefore, it should be considered alongside other peaks in the lipid assignment. To clarify this discrepancy, future experiments should be conducted on fresh specimens or even *in vivo* to validate the effect of protein–lipid interaction.

Another factor to consider in regard to lipids is the deparaffinization process, which is a critical step in tissue sample preparation for Raman spectroscopy, removing prominent paraffin peaks. It can induce changes in Raman spectra due to the removal of the paraffin matrix, affecting peak intensity and shape. Consequently, it is essential to consider the effects of deparaffinization on Raman spectra, and we will be taking measures to minimize potential interference in the future, especially for accurate identification of lipid bands, crucial for reliable spectroscopic analysis.

## 4 Discussion

Whether cause or effect, biochemical alterations of tissue components are fundamental to most, if not all, pathological states, including FCD-induced focal epilepsy.^29^ Raman spectroscopy has the potential to detect these critical alterations with high sensitivity and specificity. Focal cortical dysplasia has an intrinsic epileptogenicity, the cause of which remains unresolved. Here, it was hypothesized that differences in the Raman spectral profiles of FCD type II would be sufficient to detect changes in the biomolecular composition of cells and/or metabolic byproducts when compared with normal brain cells. Previous work by other authors supported this hypothesis: Anand et al.^14^ used a Raman/fluorescence spectroscopy fiber optics probe to study the composition of seven assorted FCD specimens: three were FCD type Ib, three were FCD type IIa, and one was FCD type IIIb. They were able to distinguish FCD from three normal cortex specimens with a sensitivity of 100% and a specificity of 70%. This work was encouraging despite a low number of samples and a diversity of FCD types that prevented definite conclusions to be reached.

FFPEs are vital steps for keeping tissue preserved in a way that allows precise microscopic images to be obtained. Frozen sections may be obtained; however, sections are often torn during the cryosectioning process, making it more difficult to localize Raman regions with stained regions. FFPE can induce changes to molecular bonds in a sample. However, this protocol was applied to both FCD and normal cortex tissues, so molecular bonds will be affected to the same extent.

Deparaffinization is a crucial step in tissue sample preparation for Raman spectroscopy. This removes prominent paraffin peaks which can otherwise overwhelm biological signals.^30^ By comparing our spectra to the known spectra of paraffin, we have determined that paraffin is removed, as the key paraffin peaks at 1462, 1063, and $1133\;{\mathrm{cm}}^{-1}$ are not apparent. Although deparaffinization may remove some lipids, this step leaves lipids bound to proteins untouched.^31^^,^^32^ Myelin (the lipid found wrapped around neurons) is wrapped in layers and bound to proteins such as the myelin basic protein.^33^ Again, this step was applied to both FCD and normal cortex tissues.^33^

The goal of our study was to use Raman microspectroscopy on a larger number of FCD specimens that were all type II and to build classifiers distinguishing cells from dysplastic tissue with respect to normal brain cells. Our aim was also to acquire a sufficiently large number of measurements to test whether or not Raman spectroscopy could distinguish among different FCD types, i.e., types IIa and IIb. When comparing FCD type II cells to the normal cortex, Raman spectroscopy revealed several discriminatory vibrational bonds, elucidating some aspects of the biomolecular origin of those changes (Table 1). The classification results associated with machine learning models confirmed FCD type II could be distinguished from the normal brain with an accuracy of 96%, a sensitivity of 100%, and a specificity of 95%. Discrimination of FCD types IIa and IIb was also achieved, albeit with lower performance figures. An accuracy of 92%, a sensitivity of 100%, and a specificity of 86% were achieved, demonstrating Raman microspectroscopy detected molecular compositions of different histopathological subtypes.

Currently, even the most advanced neuroimaging and electrophysiology tests, as part of the presurgical evaluation, can only provide a reasonable localization of the epicenter of the EZ along with a “best estimate” of its borders. Even with the greatest presurgical confidence that the EZ has been properly identified and supported by state-of-the-art intraoperative adjuncts, such as iMRI and Ecog, the neurosurgeon will not know exactly where the FCD type II pathology stops and the normal, healthy brain begins. Raman spectroscopy holds promise to be the next frontier to optimize (i.e., maximize) the surgical resection of epileptogenic tissue (just as has been shown with brain tumors) without transgressing into eloquent nervous tissue.

## 5 Conclusion

This proof-of-concept study demonstrated the potential of *ex vivo* Raman spectroscopy as a valuable tool for gathering crucial information from focal cortical dysplasia type II tissue samples. In addition, this technique provides insights into the multiple biochemical alterations within dysplastic tissues, which may contribute to the underlying mechanisms of epileptogenesis. To further advance this line of investigation, the transition to *in vivo* *in situ* assessment using a handheld fiber optics probe in a surgical setting is recommended to evaluate the real-time capability of this technology in distinguishing FCD type II from normal brain tissue. Our laboratory has developed a Raman probe and is actively conducting *in vivo* studies during epilepsy surgeries, marking a significant advancement in this research area. This Raman probe is poised to become a crucial tool in the existing multimodal approach for detecting and safely resecting focal cortical dysplasia (FCD), thereby enhancing postoperative seizure and functional outcomes for patients with focal epilepsy. In addition, there is a growing need for Raman imaging in other medical fields, including its potential applications in detecting and studying gastric cancer,^34^^,^^35^ brain cancer,^36^ and breast cancer,^37^ which could lead to significant advancements in the diagnosis and treatment of other diseases.

## Data Availability

The code and script package for our data processing is hosted on the Python Package Index as an open-source project under the name ORPLIB and is distributed under the MIT License, ensuring free usage and modification by the community. The complete source code is transparently available on GitHub at https://github.com/mr-sheg/orpl. We encourage researchers, developers, and users to explore, contribute, and report any issues via the GitHub repository. Comprehensive documentation and example scripts are also provided to facilitate ease of use. By maintaining this project as open source, we aim to foster collaboration, ensure reproducibility, and continually improve the package based on community feedback.
